# Reciprocal effect between non-suicidal self-injury and depressive symptoms in adolescence

**DOI:** 10.3389/fpubh.2023.1243885

**Published:** 2024-01-11

**Authors:** Rui Hu, Li-Li Peng, Yu Du, Yi-Wei Feng, Lin-Shen Xie, Wei Shi, Peng Jia, Li-Hua Jiang, Li Zhao

**Affiliations:** ^1^West China School of Public Health and West China Fourth Hospital, Sichuan University, Chengdu, China; ^2^West China-PUMC C.C. Chen Institute of Health, Sichuan University, Chengdu, China; ^3^Department of Emergency and Critical Care Medicine, West China School of Public Health and West China Fourth Hospital, Sichuan University, Chengdu, China; ^4^Duke Kunshan University, Suzhou, China; ^5^Department of Occupational Health, West China School of Public Health and West China Fourth Hospital, Sichuan University, Chengdu, China; ^6^Institute for Disaster Management and Reconstruction (IDMR), Sichuan University, Chengdu, China; ^7^International Institute of Spatial Life Course Epidemiology (ISLE), Wuhan University, Wuhan, China; ^8^Teaching and Research Section of General Practice, The General Practice Medical Center, West China Hospital of Sichuan University, Chengdu, China

**Keywords:** non-suicidal self-injury, depressive symptoms, adolescence, follow-up study, COVID-19

## Abstract

**Background:**

Non-suicidal self-injury (NSSI) is a common psychological and behavioral problem among adolescents. The COVID-19 pandemic has had a significant impact on people’s mental health. To date, few studies have documented the temporal changes in adolescents’ psychological status during the pandemic, as well as the impact of large-scale public health intervention strategies. This study contributes to the existing evidence on the subject.

**Methods:**

Participants were 6,023 adolescents aged 10 years and older, with data from two waves of longitudinal surveys, including data for a 7-month interval before and during the pandemic. A cross-lagged model was used to test the bidirectional relationship between NSSI and depressive symptoms in adolescents; logistic regression analysis was used to explore the predictors of NSSI implementation in adolescents with depressive symptoms.

**Results:**

In this study, 32.69% participants reported depressive symptoms at baseline and 34.27% at follow-up; 44.34% participants with depressive symptoms reported NSSI at baseline and 53.44% at follow-up. The duration of the online class, depressed affect, and somatic and related activity were the risk factors for NSSI; sleep duration and positive mood were the protective factors. The lag effect of depression symptoms on NSSI is significant, and so is NSSI on depressive symptoms.

**Conclusion:**

During the COVID-19 pandemic, adolescents’ mental health has worsened, resulting in an increase in the prevalence of NSSI among those with depressive symptoms compared to pre-pandemic levels. Early screening for depression is crucial in preventing or decreasing NSSI in adolescents.

## 1 Introduction

The outbreak of the COVID-19 pandemic has presented significant challenges to people’s mental well-being (1). After the outbreak, countries, including China, implemented lockdown or semi-lockdown measures, disrupting the normal routines of daily life. The uncertainty and concerns surrounding contracting the virus have intensified the psychological health crisis (2, 3). A systematic review of studies on COVID-19-related psychological distress in countries such as China, Italy, and the United States revealed high prevalence rates of anxiety (6.3 to 50.9%), depression (14.6 to 48.3%), post-traumatic stress disorder (7 to 53.8%), psychological distress (34.4 to 38%), and stress (8.1 to 81.9%) among the general population (4).

The global mental crisis coronavirus disease 2019 (COVID-19) had created a challenging situation for all, especially adolescents. Adolescence is characterized by prominent relationships with peers. Research suggests that friends provide companionship and social and emotional support as adolescents pursue independent development (5). However, home isolation, maintaining social distancing, and closing schools hindered peer interactions, especially school closures, and reduced students’ accessibility to external psychosocial protective resources during COVID-19. Therefore, it is necessary to study the influence of the COVID-19 pandemic on adolescent mental health. Adolescence is a time when the incidence of psychological problems is high, with approximately 50% of psychological disorders, including anxiety, depression, and other negative feelings, appearing before the age of 14 years (6, 7). Data on adolescents before the COVID-19 pandemic in China suggest that 19.9% of adolescents had already experienced depressive symptoms, and 13.99% had experienced anxiety disorders (8). A systematic review and meta-analysis revealed that 28.6 and 25.5% of adolescents experienced symptoms of depression and anxiety, respectively, during the COVID-19 pandemic (9). The COVID-19 pandemic has affected the mental health of adolescents.

Adolescence is also a critical period for mental health issues and risky behaviors such as non-suicidal self-injury (NSSI), which refers to the direct intentional destruction of one’s body without suicidal intent, including cutting, scratching, burns, stabbing, and biting, but is not life-threatening (10, 11). Adolescents are at a high risk of NSSI, with a lifetime prevalence of 17.2% (12). The prevalence of NSSI in Chinese adolescents was 21.9% (13) and 27.4% among middle school students (14). Some data show that the prevalence of NSSI among Chinese adolescents has been on the rise in recent years (15). NSSI not only causes harm to one’s body, but also adversely affects family and interpersonal relationships, and NSSI is also a risk factor for subsequent suicidal behavior (16). There is evidence that NSSI is associated with a range of psychological difficulties, including depression, anxiety, and post-traumatic stress disorder (17). A meta-analysis of factors related to NSSI among Chinese adolescents showed that adolescents with mental health problems were more than 1.5 times more likely to develop NSSI than those without mental health problems (18). A systematic evaluation of 39 studies pointed out that, based on adolescents’ depressive symptoms, one can predict their chances of committing NSSI in the future (19). A longitudinal study with a sample of 813 Chinese adolescents also showed that higher levels of depressive symptoms were associated with an increase in NSSI one year later (20). However, the long-term effects of depressive symptoms on NSSI in adolescents remain unknown in the context of COVID-19.

The WHO emphasized the need for people to study the psychological impact COVID-19 has caused (21). Thus far, there is a relatively small record of longitudinal change in adolescents’ psychological status during the COVID-19 pandemic as well as with the implementation of large-scale public health intervention methods (22–24). To investigate the influence of depressive symptoms on NSSI among adolescents and the occurrence rate of NSSI among adolescents with depressive symptoms, we performed this follow-up study. Furthermore, to assess the bidirectional association between depressive symptoms and NSSI to provide more evidence for a follow-up study of NSSI in Chinese adolescents. Therefore, we hypothesized that during COVID-19, there will be an increase in NSSI behaviors and the incidence of depressive symptoms. In addition, psychological health among adolescents deteriorated, and the prevalence of NSSI among adolescents with depressive symptoms increased compared with that before the pandemic.

## 2 Methods

### 2.1 Design and participants

Five schools in Chengdu were selected for this study using multi-stage stratified cluster sampling. Stage 1: The city of Chengdu was divided into high, medium, and low levels according to the level of economic development, with one district randomly selected from each level; Stage 2: One school was randomly selected in each district (county); and Stage 3: all students in the school were included in the study. In this way, we selected one school in the center of Chengdu City and two schools in each of the two suburban counties to the north and south. Among these schools, two schools included both primary and secondary students, two were primary school and one was middle school. All students in the selected schools were included in the survey. Before conducting the targeted school surveys, the researchers coded all students in all schools individually and created survey manuals to train classroom teachers and quality controllers (QC). Then the school was organized to conduct the questionnaire survey and all respondents were grouped into classes, each class was assigned a classroom teacher and a QC. The class teacher led the students to fill in the questionnaire and explained the content of the questionnaire according to the student’s cognitive ability. When the classroom teacher encounters a student question that cannot be answered, the QC will explain.

There are two waves of data collection. The Wave 1 survey was carried out between December 23, 2019, and January 13, 2020 (before the pandemic, at baseline). We did an on-site survey and distributed questionnaires from December 23, 2019 until the end of December. We collected questionnaires and processed data from the beginning of January through January 13, 2020. This was before the outbreak of COVID-19 in Wuhan, China, and the school closure. A year following the baseline survey, a wave 2 survey was planned. It was, however, carried out 6 months ahead of schedule to record the immediate impact of the COVID-19 pandemic. So, from June 16 to July 8, 2020 (during the pandemic, at follow-up), wave 2 data was obtained when schools reopened following the COVID-19 outbreak.

We received 6,190 and 6,654 completed questionnaires in Wave 1 and Wave 2 surveys, respectively, from adolescents aged 10 and up. Finally, 6,023 valid respondents were included after excluding questionnaires with more missing (variable missing more than 20%), not answering attentively, and inconsistent personal information in the two surveys.

### 2.2 Ethics approval and consent to participate

The study was conducted by the Declaration of Helsinki. The schools, students, and parents participating in the survey provided written informed consent, whereas the children provided parental informed consent before inclusion in the study. The study protocol was approved by the Medical Ethics Committee of Sichuan University (Ethics No. K2020025).

### 2.3 Measuring tools

#### 2.3.1 Deliberate self-harm inventory, DSHI

The Deliberate Self-Harm Inventory (DSHI) was used to measure the NSSI. Gratz (2) created and verified a scale that Lundh et al. (25, 26) reduced and shortened. The Chinese version of the DSHI has also been proven to have high reliability and validity when applied to Chinese children and adolescents (27, 28). Adolescents were asked if they had ever experienced self-injury, including cuts, burns, scratches, bites, stabbing, or other types of NSSI. To measure the existence or absence of NSSI behaviors, a scoring system was employed, and the frequency of each NSSI was designed as 0 points for “never,” 1 point for “1 time,” 2 points for “2 times,” and 3 points for “3 times or more.” The NSSI scores were categorized into dichotomous “yes” and “no” variables. “Without” refers to a cumulative total score of 0, and “with” refers to a cumulative score of ≥1 for each of the 8 NSSI modalities. Cronbach’s α for the surveyed sample was 0.875.

#### 2.3.2 Center for epidemiologic studies depression scale for children, CES-DC

The Center for Epidemiological Studies for Children (CES-DC) (29) was used to assess depressive symptoms in participants during the past week. Dimensionality and factorial invariance were also investigated (30). Radloff (31) designed the scale. In addition to being widely used worldwide, the CES-DC has been applied to Chinese adolescents with good reliability and validity (32). The CES-DC includes four dimensions: depressed affect (8 items), positive affect (4 items), somatic symptoms and related activity (6 items), and interpersonal (2 items). Each item is answered on a four-point scale (0 = very little or no time, 1 = some or a little time, 2 = moderate or most of the time, and 3 = most or all of the time). A total score of 15 shows no depression symptoms, a total score of 16 to 27 suggests the possibility of depressed symptoms, and a total score greater than 27 indicates confirmed depressive symptoms. Participants who had both the likelihood of and definite depression symptoms were deemed to have depressive symptoms (33). The Cronbach’s α of this study is 0.84.

#### 2.3.3 Demographics and COVID-19 infection history

The demographic data consists of nine items: age (year), gender (boy or girl), self-evaluation of caregiver relationship (Do you have a good relationship with your caregivers? “1 “means very bad,” 10 “means very good”), place of residence (urban and rural), sleep duration, exercise time, and duration of online classes during the pandemic (From the COVID-19 Pandemic to the Back-to-School Period, how long were your daily sleep/exercise time and duration of online classes respectively?), history of COVID-19 infection (Have you or your family members been infected by COVID-19?).

### 2.4 Data analysis

SPSS (version 22.0) and Mplus (version 7.3) were used to analyze the data. Descriptive statistics include mean and standard deviation (SD) for continuous variables, whereas categorical variables include frequency and percentage. *T*-test, Chi-square, and logistic regression models were used to analyze the connection between depression symptoms and NSSI. Data that do not follow a normal distribution are represented by the median and quartile, replace the t-test with the Wilcoxon signed rank sum test. Cross-lagged analysis was utilized to explore the bidirectional relationship between depressive symptoms and NSSI. To assess overall model quality and path significance, a variety of fit indices were used, including χ^2^/df (degrees of freedom), Root Mean Square Error of Approximation (RMSEA), CFI (Comparative Fit Indices), and TLI (Tucker Lewis Index). When χ2/df is less than 5.0, the CFI and TLI are all greater than 0.95 and the RMSEA is less than 0.05, as a good fit, the longitudinal cross-lagged route model is supported (34). Furthermore, the model demonstrates that the proposed model can accurately reproduce the observed longitudinal data. All statistical tests used were two-sided, and a *p* value of less than 0.05 was considered statistically significant.

## 3 Results

### 3.1 Demographic profile of study participants

The study used two waves of data-complete samples, with 6,023 adolescents aged 10 years and older included in the survey. The oldest was 17(Mean age = 11.63, SD = 1.54, at Wave 1). 3,121 boys (51.82%) and 2,902 girls (48.18%) were included. 5,048 adolescents (89.78%) assessed their relationship with caregivers as good (≥ 5 points), and 615 adolescents (10.21%) assessed such relationship as bad (< 5 points). 3,679 (61.08%) of all adolescents included resided in urban areas, while the rest 2,344 (38.92%) lived in rural regions. During the pandemic, adolescents’ average daily sleep duration was 8.64 (1.50) hours, their average physical exercise duration was 1.55 (1.41) hours, and their average online class duration was 5.64 (2.92) hours. COVID-19 infected 111 people (1.84%) and 732 households (12.15%) lost their jobs.

After performing a normality test, we found that the NSSI data did not meet the requirements. Therefore, logarithmic transformation was performed on the NSSI data. At baseline, 29.61% of boys and 27.65% of girls reported NSSIs; at follow-up, the statistics were 33.49 and 35.73%, respectively. Among students with depressive symptoms, the occurrence rate of NSSI was 44.34 and 53.44%, respectively, and the occurrence rate of NSSI increased in both surveys. The occurrence of NSSI decreased among students without depressive symptoms (Table 1).

**Table 1:** Occurrence of NSSI in adolescents with different demographic characteristics (*N* = 6,023).

Demographic characteristics	Number of people		Number of NSSI occurrences (Incidence%)		χ^2^/T		*p*	
	W1	W2	W1	W2	W1	W2	W1	W2
Age	6,023							
Gender					10.54	45.49	<0.01	<0.01
Boy	3,121		924 (29.61)	863 (27.65)				
Girl	2,902		972 (33.49)	1,037 (35.73)				
Depressive symptoms					568.61	697.03	<0.001	<0.01
Yes	1969	2064	1,023 (44.34)	1,103 (53.44)				
No	4,054	3,959	873 (21.53)	797 (20.13)				
Self-perception of relationship with caregivers					0.05	267.35	>0.05	<0.01
≥5points	5,452	5,407	1714 (31.44)	1,527 (28.04)				
<5points	571	616	182 (31.87)	373 (60.55)				
Place of residence					6.30	26.53	<0.01	<0.01
City or town	3,679	1,114 (30.28)	1,070 (29.08)					
Rural area	2,344	782 (33.36)	830 (35.41)					
COVID-19 infection history				/		0.38	/	>0.05
Yes	/	111	/	38 (34.23)				
No	/	5,912	/	1862 (31.50)				
Unemployed due to COVID-19				/		6.09	/	<0.01
Yes	/	732	/	260 (35.52)				
No	/	5,291	/	1,640 (31.00)				

W1 = At baseline; W2 = At follow-up.

### 3.2 The alteration of NSSI and depressive symptoms at baseline and follow-up

A paired t-test of depressive symptoms was conducted to assess the differences between baseline and follow-up. The Wilcoxon signed-rank sum test was used for NSSI. The NSSI scores showed a statistically significant difference between the baseline and follow-up periods, and the same was observed for depression symptoms, with an increase in scores compared to the previous period (*p* < 0.01) (Table 2).

**Table 2:** The alteration of NSSI and depressive symptoms at baseline and follow-up (*N* = 6,023).

Grouping	At baseline	At follow-up	Wilcoxon signed the rank sum test	
	M ± SD/Median (Quartile)	M ± SD/Median (Quartile)	*T*	*p*
NSSI	0 (0, 1.26)	0 (0, 2)	4.43	<0.01
Depressive symptoms	14.15 ± 10.34	14.55 ± 10.90	−3.09	<0.01

M, Mean; SD, Standard Deviation.

McNemar et al. suggested an increase in the incidence of NSSI in adolescents (37.71 to 38.32%); however, the difference was not statistically significant (*p* > 0.05). The occurrence rate of depressive symptoms among adolescents was higher than that at baseline (32.69–34.27%, *p* < 0.01) (Table 3).

**Table 3:** The alteration of the occurrence rate of depression in adolescents at baseline and follow-up.

W2	W1		Total number(%)	χ2	*p*
	NSSI/Depressive symptoms	Non-NSSI/Non-depressive symptoms			
NSSI	1,609	699	2,308 (38.32)	0.94/5.53	0.33/<0.05
Non-NSSI	662	3,053	3,752 (62.29)		
Depressive symptoms	1,201	863	2064 (34.27)		
Non-depressive symptoms	768	3,191	3,959 (65.73)		
Total number(%)	2,271 (37.71)/1969 (32.69)	3,715 (61.68)/4,054 (67.31)	6,023		

### 3.3 The alteration of depression symptoms scores in different dimensions at baseline and follow-up

The test of normality for the four dimensions of depressive symptoms showed that the vast majority of points could be distributed on a straight line with a clear linear trend, and the continuous data could be considered as obeying a normal distribution. Data analysis using independent samples T-test. According to the analysis from the independent sample test, differences exist in the scores of depressed affect, positive affect, somatic and related activity, and interpersonal relationships between the NSSI group and the Non-NSSI group (*p* < 0.01) (Table 4).

**Table 4:** The alteration of depression symptoms scores in different dimensions at baseline and follow-up.

variables	Group	W1				W2			
		Mean	SD	*T*	*p*	Mean	SD	*T*	*p*
Depressed affect	Non-NSSI group	2.67	3.64	−32.04	<0.001	2.62	3.82	−34.29	<0.01
	NSSI group	6.62	5.82			7.02	6.04		
Positive affect	Non-NSSI group	5.74	3.76	−11.44	<0.001	5.93	3.86	−12.65	<0.01
	NSSI group	6.90	3.35			7.22	3.22		
Somatic and related activity	Non-NSSI group	2.41	2.87	−29.16	<0.001	2.38	3.06	−31.56	<0.01
	NSSI group	5.11	4.16			5.44	4.29		
Interpersonal	Non-NSSI group	0.59	1.18	−22.44	<0.001	0.56	1.18	−24.85	<0.01
	NSSI group	1.46	1.77			1.53	1.82		

### 3.4 Binary logistic regression analysis revealing effect on NSSI

Taking whether adolescents commit NSSI as a dependent variable while considering gender, age, depression measurement factors, duration of sleep, duration of exercise, duration of online class, self-perception of relationship with caregivers, residence, and whether unemployed due to COVID-19 as the independent variables, a binary logistic regression analysis (forward: LR method) was conducted. The introduction level was 0.05 and the exclusion level was 0.1. The statistical results showed that among adolescents, females, age, duration of the online class, self-perceived poor relationship with caregivers, depression mood, somatic and related activity were the risk factors for NSSI (or > 1, β > 0), living in rural area, sleep duration and positive mood was the protective factor (or < 1, β < 0) (Table 5).

**Table 5:** Results of binary Logistic regression analysis revealing effect on NSSI.

Factors	B	S.E.	Wald	Significance	Exp (B)	95% C. I	
						Lower	Upper
Constant	−3.49	0.40	76.37	<0.01	0.03		
Gender = female	0.34	0.07	26.73	<0.01	1.41	1.24	1.60
Age	0.05	0.02	5.66	0.017	1.06	1.01	1.10
sleep duration	−0.09	0.02	14.68	<0.01	0.92	0.87	0.96
Duration of online classes	0.27	0.01	515.23	<0.01	1.31	1.28	1.35
Place of residence = rural area	−0.30	0.07	20.18	<0.01	0.74	0.65	0.84
Self-perception of relationship with caregivers = Bad	0.53	0.11	23.76	<0.01	1.70	1.37	2.10
Depression mood	0.11	0.0.01	87.95	<0.01	1.12	1.09	1.15
Positive mood	−0.06	0.01	37.23	<0.01	0.94	0.93	0.96
Somatic and related activity	0.07	0.02	21.13	<0.01	1.08	1.04	1.11

### 3.5 Longitudinal, bilateral relations between NSSI and depressive symptoms

A cross-lagged model was used to explore the longitudinal relationship between depressive symptoms and self-injury, with gender and age as control variables. Variables were non-normal data and parameter estimation was performed using the MLM, provided a good fit (χ^2^/df = 2.74<3, CFI = 0.996>0.95, TLI = 0.993>0.95, RMSEA = 0.017<0.05).

**Figure 1:**
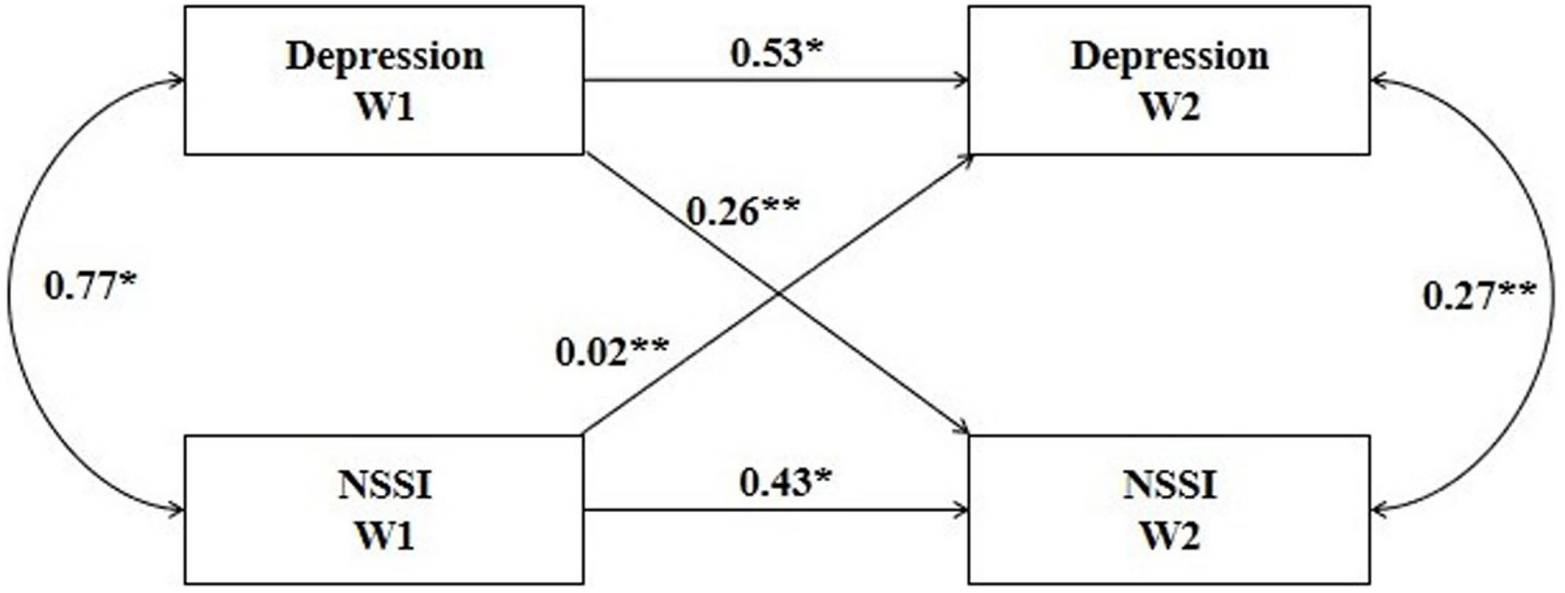
The cross-lagged model between NSSI and depressive symptoms. Using sex and age as control variables, the two-way arrow in the chart indicates the result of correlation analysis, with the data of correlation coefficient; the one-way arrow indicates the result of path analysis, with the data of standardized regression coefficient (*β*). *p < 0.05. **p < 0.01. Depression W1: depressive symptoms at baseline; Depression W2: depressive symptoms at follow-up; NSSI W1: NSSI behavior at baseline; NSSI W2: NSSI behavior at follow-up.

Figure 1 illustrates the normalized coefficients for all model paths. At seven-month intervals, NSSI and depression symptoms were associated, with correlation coefficients of 0.77 (*p* < 0.05) and 0.27 (*p* < 0.01), respectively. A steady association was found between pre-pandemic NSSI and post-pandemic NSSI, implying that pre-existing NSSI can predict subsequent (β = 0.43, *p* < 0.05). The same was true for depression symptoms (β = 0.53, *p* < 0.05). There was a significant lag effect of adolescents’ depressive symptoms on NSSI, meaning that the deeper the adolescents were depressed, the more frequent their NSSI was, controlling for the adolescents’ baseline NSSI (β = 0.26, *p* < 0.01). There was also a lag effect of NSSI on depressive symptoms (β = 0.02, *p* < 0.01). However, depressive symptoms were more predictive.

## 4 Discussion

To our knowledge, the hypothesis that “an increase in the prevalence of depressive symptoms and prevalence of NSSI with depressive symptoms” was confirmed. Adolescents had relatively high rates of depressive symptoms (32.69 to 34.27%) and NSSI with depressive symptoms (44.34 to 53.44%). These findings enrich theoretical research on teenage mental health and NSSI in the context of the COVID-19 pandemic. First, this study did not find an increased incidence of NSSI during the COVID-19 pandemic. Nevertheless, the NSSI scores showed statistically significant differences between baseline and follow-up. Prolonged exposure to the uncertainty of infection may trigger vulnerability in adolescents and lead to changes in NSSI from the pre-pandemic period to several months later. There was an increased incidence of NSSI among adolescents with depressive symptoms. This suggests that adolescents with preexisting mental distress perceived the negative events that occurred during COVID-19 as more stressful, which may have contributed to their increased likelihood of participating in NSSI (6, 35). Specifically, adolescents with prior depressive symptoms, relatively high rates of internalizing problem behaviors, and poorer emotional regulation experienced more COVID-19-related stress and were at a higher risk of engaging in NSSI.

NSSI and depressive symptoms in adolescents before COVID-19 predicted these issues after COVID-19. Studies have shown that underlying psychological and psychiatric problems usually occur after disasters, the most common being depression (36, 37). If this theory were applied to the pandemic, we could infer that adolescents who had never experienced mental illness before COVID-19 may experience psychiatric symptoms such as depression, distress, stress, and anxiety. Groups that had previously suffered psychological distress may have exacerbated preexisting mental and emotional distress (38, 39). However, adolescents are emotionally vulnerable and easily stimulated by negative life events to experience emotional outbursts, which, in turn, produce several negative emotions. When there is an inability to regulate negative emotions, they may resort to NSSI to transfer them. According to the experiential avoidance model proposed by Chapman et al., NSSI can provide temporary relief from adolescents’ negative emotions. Nevertheless, when adolescents are again exposed to negative emotional stimuli, they tend to use NSSI for relief, which continuously facilitates the occurrence of NSSI (40). Based on this theory, adolescents with pre-existing vulnerabilities may be more stressed in the face of the COVID-19 pandemic and are at a higher risk of engaging in NSSI. This study also found that NSSI was a predictor of depressive symptoms. NSSIs such as cuts or burns may be a coping mechanism for those experiencing emotional distress or mental health problems. These behaviors may provide temporary relief; nonetheless, they do not address the root cause of distress and can instead lead to feelings of guilt, shame, or despair—common symptoms of depression. Thus, adolescents participating in NSSI are more likely to experience depressive symptoms.

Data from this study found that adolescents who had engaged in the NSSI scored higher on all dimensions of depressive symptoms than those who had not participated in the NSSI; the highest and lowest scores were for depressed affect and interpersonal, respectively. Depression symptoms are primarily defined as emotional problems characterized by a predominantly depressed state of mind, ranging from mildly negative emotional experiences to severe mood disorders (41). In turn, depressive symptoms are associated with chronic dopamine downregulation, which can be accompanied by psychomotor inhibition, and may have a synergistic effect on clinical deficits in adolescents’ affective, cognitive, and motor behaviors, leading to unresponsiveness, reduced speech and movement, reluctance to communicate with others, and enjoyment of solitude, all of which are harmful to adolescents’ interpersonal development (42, 43). Regression analyses revealed that depressed mood and somatic and related activities were risk factors for NSSI. Adverse life events that are usually capable of directly causing negative emotional experiences are risk factors for prompting and maintaining NSSI in adolescents (44).

Adolescents’ self-perceptions of their relationship with their caregivers are predictors of NSSI, and adolescents who perceive themselves as having a poor relationship with their caregivers are more likely to engage in NSSI. Studies have shown that dysfunctional family environments increase the risk of NSSI and that parental conflict and parent–child conflict are associated with a range of psychological problems in adolescents (45, 46). The duration of online classes during the pandemic was also a predictor of NSSI, and the fact that parents had to support their children’s homeschooling while working at home may have increased the risk of parent–child conflict. Past theories on the psychological effects of an approaching disaster have predicted how the place of residence will affect mental health, namely “psychological typhoon eye” (47). The results of this study support the psychological typhoon-eye effect, as adolescents living in rural areas have a lower risk of developing NSSI than those living in urban areas, regardless of whether or not these adolescents have depressive symptoms. The potential explanation was a decrease in social contact. During the pandemic, adolescents have been forced to reduce their social contacts, and involuntary reductions in social contact may cause more suffering among socially active urban adolescents than among rural adolescents. NSSI may also increase as a result of limited social contact, especially among those who are mentally vulnerable. Gender was one of the predictors of adolescent NSSI during the pandemic, consistent with previous findings (48).

Positive emotions were also found to be positive predictors. Evidence from previous studies suggests that disasters and pandemics can stimulate social cohesion and solidarity (49, 50). For example, after the outbreak of severe acute respiratory syndrome in Hong Kong in 2003, residents’ sense of belonging to friends and family increased (51, 52). Therefore, we believe that, in the context of the pandemic, the widely shared experience of combating the pandemic may enhance social cohesion and intimacy, which may change adolescents’ views on death and health. Adolescents who develop psychological disorders may experience a climate of social support during difficult pandemic prevention. During the COVID-19 pandemic, people supported the government’s strategies and measures to prevent and control the pandemic, the community provided help for survival and medical supplies for the sealed control area, and healthcare workers stood firm on the frontline of the fight against the pandemic. Such positive feedback may promote adolescents’ psychological functioning and lead to positive psychological changes. This study also found that sleep duration has an important protective effect against NSSI. Previous studies have identified poor sleep quality and frequent nightmares as important risk factors for NSSI. A study of 223 adolescents with self-harming behaviors found that 2% had serious sleep problems (53). However, adolescents with sleep disorders usually experience NSSI mediated by mood disorders. Shorter sleep duration is directly related to depression (54). Lack of sleep can lead to decreased mood regulation, and chronic sleep deprivation may increase the risk of depression and trigger NSSI.

To conclude, adolescents with NSSI have worse depressive symptoms, and adolescents with depressive symptoms are at higher risk for NSSI; they should be the primary group for attention and intervention. Timely attention should be paid to the negative emotions of adolescents, and screening for depression, as well as their assessment and treatment, should be conducted. For adolescents with NSSI, it is important to intervene as early as possible and provide effective psychological support from schools, families, and society to help them realize the value of life. Finally, it is necessary to develop the mental toughness of young people and improve their abilities to withstand stress and adversity.

## 5 Limitations

This study had several limitations. To begin, the duration of the follow-up research was 7 months, which is short but fair because we collected data before and after the school shutdown in Chengdu. Furthermore, the Center for Epidemiological Studies for Children (CES-DC) instrument is a self-report screening scale rather than a diagnostic tool, which may be a major limitation. It assesses the intensity of depressive symptoms over the previous week, which is also a short timeframe for making a diagnosis. Finally, as this study collected data using a self-administered questionnaire, there may be some confounding issues, such as remembrance bias and report bias, which could have affected the stability of our study’s longitudinal connection. Further advancements are required in future research. Additionally, the sampling technique for this study was divided geographically, while the discussion was held in urban and rural areas. The use of sample data is insufficient, and there may be conflicting data.

## 6 Conclusion

Reciprocal effects between depression symptoms and NSSI among Chinese adolescents were found in our study. The increasing trend in the incidence of depressive symptoms and NSSI with depressive symptoms before and during the pandemic, demonstrates that COVID-19 may have had some impact on the mental health of adolescents. The current study also reveals that sleep duration and positive emotions are protective factors for NSSI. Sleep duration is essentially a modifiable influence on depressive symptoms and can also alter the NSSI. Positive emotion, as an intrinsic psychological trait, suggests that future research could already be conducted from a positive psychology perspective, focusing on the positive traits of adolescents.

## Data availability statement

The datasets generated and/or analyzed during the study are not publicly available due to reasons of sensitivity e.g. human data. Anonymized data can be made available from the corresponding author on reasonable request.

## Ethics statement

The studies involving humans were approved by The study protocol was approved by the Medical Ethics Committee of Sichuan University (Ethics No. K2020025). The studies were conducted in accordance with the local legislation and institutional requirements. Written informed consent for participation in this study was provided by the participants' legal guardians/next of kin.

## Author contributions

RH: writing original draft, writing- reviewing and editing, methodology, data curation, investigation, and formal analysis. L-LP: writing - original draft, writing- reviewing and editing, and data curation. YD, Y-WF, L-SX, and PJ: writing - review & editing. WS: writing - review & editing, methodology, language editing. L-HJ: writing - review & editing, supervision. LZ: conceptualization, methodology, project administration, and funding acquisition. All authors contributed to the article and approved the submitted version.
