# Corrigendum: Reciprocal effect between non-suicidal self-injury and depressive symptoms in adolescence

**DOI:** 10.3389/fpubh.2025.1502519

**Published:** 2025-03-10

**Authors:** Rui Hu, Li-Li Peng, Yu Du, Yi-Wei Feng, Lin-Shen Xie, Wei Shi, Peng Jia, Li-Hua Jiang, Li Zhao

**Affiliations:** ^1^West China School of Public Health and West China Fourth Hospital, Sichuan University, Chengdu, China; ^2^West China-PUMC C.C. Chen Institute of Health, Sichuan University, Chengdu, China; ^3^Department of Emergency and Critical Care Medicine, West China School of Public Health and West China Fourth Hospital, Sichuan University, Chengdu, China; ^4^Duke Kunshan University, Suzhou, China; ^5^Department of Occupational Health, West China School of Public Health and West China Fourth Hospital, Sichuan University, Chengdu, China; ^6^Institute for Disaster Management and Reconstruction (IDMR), Sichuan University, Chengdu, China; ^7^International Institute of Spatial Life Course Epidemiology (ISLE), Wuhan University, Wuhan, China; ^8^Teaching and Research Section of General Practice, The General Practice Medical Center, West China Hospital of Sichuan University, Chengdu, China

**Keywords:** non-suicidal self-injury, depressive symptoms, adolescence, follow-up study, COVID-19

In the published article, there was an error. Some statements were accidentally deleted while revising the manuscript and there were accidental transcription errors during the revision.

A correction has been made to **1 Introduction**, paragraph three. This sentence previously stated:

“Adolescents are at a high risk of NSSI, with a lifetime prevalence of 17.2% (12). Adolescents are at a high risk of NSSI, with a lifetime prevalence of 17.2% (13) and 27.4% among middle school students (14).”

The corrected sentence appears below:

“Adolescents are at a high risk of NSSI, with a lifetime prevalence of 17.2% (12). The prevalence of NSSI in Chinese adolescents was 21.9% (13) and 27.4% among middle school students (14).”

A correction has been made to **1 Introduction**, paragraph three. This sentence previously stated:

“A meta-analysis of factors related to NSSI among Chinese adolescents showed that adolescents with mental health problems were more than 1.5 times more likely to develop NSSI than those without mental health problems (18). A meta-analysis of factors related to NSSI among Chinese adolescents showed that adolescents with mental health problems were more than 1.5 times more likely to develop NSSI than those without mental health problems (19). ”

The corrected sentence appears below:

“A meta-analysis of factors related to NSSI among Chinese adolescents showed that adolescents with mental health problems were more than 1.5 times more likely to develop NSSI than those without mental health problems (18). A systematic evaluation of 39 studies pointed out that, based on adolescents' depressive symptoms, one can predict their chances of committing NSSI in the future (19). ”

A correction has been made to **3.3 The alteration of depression symptoms scores in different dimensions at baseline and follow-up**, paragraph one. This sentence previously stated:

“According to the analysis from the independent sample test, differences exist in the scores of depressed affect, positive affect, somatic and related activity, and interpersonal relationships between the depressive group and the non-depressive group (*p* < 0.01) (Table 4).”

The corrected sentence appears below:

“According to the analysis from the independent sample test, differences exist in the scores of depressed affect, positive affect, somatic and related activity, and interpersonal relationships between the NSSI group and the Non-NSSI group (*P* < 0.01) (Table 4).”

A correction has been made to **3.5 Longitudinal, bilateral relations between NSSI and depressive symptoms**, paragraph two. This sentence previously stated:

“A steady association was found between pre-pandemic NSSI and post-pandemic NSSI, implying that pre-existing NSSI can predict subsequent (β = 0.53, p < 0.05). The same was true for depression symptoms (β = 0.43, p < 0.05).”

The corrected sentence appears below:

“A steady association was found between pre-pandemic NSSI and post-pandemic NSSI, implying that pre-existing NSSI can predict subsequent (β = 0.43, *P* < 0.05). The same was true for depression symptoms (β = 0.53, *P* < 0.05).”

The authors apologize for these errors and state that this does not change the scientific conclusions of the article in any way. The original article has been updated.

